# The Medical Guild and the B.M.A.

**Published:** 1913-09-06

**Authors:** Harding H. Tomkins

**Affiliations:** Chairman S.W. Essex Div. B.M.A. 492 Lea Bridge Road, Leyton, N.E.


### THE NATIONAL MEDICAL GUILD.

President: CHAS. BUTTAR, M.A., M.D., B.C.

Gen. Sec.: P. C. RAIMENT, M.R.C.S., L.R.C.P.

Offices: 34 Villiers Street, Strand, London, W.C.

### The Funds of Trade Unions.

*To the Editor of* The Hospital.

### The Medical Guild and the B.M.A.

*To the Editor of* The Hospital.

Dear Sir,—I have just been shown the discussion in The Hospital, August 23, between Dr. Fothergill and the Medical Guild *re* British Medical Association Special Fund, and as I was one of the Sub-Committee who submitted the Original Report I would be glad if you publish the following explanation:—

*First*, then, the idea was a "Special Fund for the *Organisation of the Profession*," not as you have it, "The B.M.A. and State Sickness Insurance."

*Second*.—The money expended out of the Fund is to include £24,000 towards expenses of Local Medical Committees (under the Act), 240 in number; four Organising Secretaries, £2,400; Legal Expenses for both mentioned, £1,000; and Travelling Expenses, £1,000. All this is for *organisation* after Collection and cannot be called "Administrative Expenses." Also the starting of new centres, £3,000 is for Organisation and nothing to do with Collection. I fail to see how the £4,000 suggested for payment of Representative Meetings and Central Committees or State Sickness Insurance Committee can be classed among "Expenditure of 13s. 4d. to collect each sovereign" as your correspondent states. These sums total £35,400, add Reserve Balance £13,000, making £48,400, and we have £1,600 which might be classed as "expenditure to collect." Any society which provides "sick pay" to the tune of £100,000 or so per annum does not call that "expenses to collect," but the "Administrative Expenses" are perhaps 5 per cent. of the total receipts. So the expenditure on *Organisation*, which is one of the *objects* of this Special Fund, cannot be classed as "expenses to collect."

As for the willingness to subscribe, the loyalty of the members, etc., of the B.M.A., Medical Guild, or any other fund or organisation would be similarly placed.

I do not agree that the "effete leaders of the profession last year" were "men incapable of taking any wide view of the situation" and "incapable of acting in the spirit of the resolutions of the representative meetings." The whole trouble arose through the supineness and parsimony of the *individual members* of the British Medical Association, for *they are the Association* and can, and did, veto and nullify all efforts to organise or unify the profession, and ultimately excused their *volte-face* in accepting the Act by the paucity of the "Defence Fund" which they themselves had failed to supply.

There is no policy in the medical profession as a whole for the simple reason that there is no means of interchange of opinion sufficient to produce anything like unanimity. To effect this I consider it is necessary first to prepare the way by mapping out the country into areas, say, ten or twelve, having a whole-time medical organiser in each, who shall acquaint himself with the ideas in his district, and acquaint every practitioner in it with the ideas of those at headquarters who are trying to form a universal medical policy for Great Britain. When these two have been compared and revised they should be expounded verbally at headquarters, notes compared, and a policy issued best suited to the majority, to be considered and adopted by all areas. This could be done expeditiously by this personal contact, with the certainty that the matters were fully and forcibly explained. By this means also men might ultimately come to realise the trade-union principles as far as they can be applied to the medical profession, and the National Medical Guild become amalgamated with the Association. Criticism is healthy, but, in my opinion, is best confined within the limits of our fraternity and not made public to all and sundry. Why not continue the meetings among practitioners and expound the advantages of trade unionism there and there only?—Yours truly,

Harding H. Tomkins, M.R.C.S., etc.,
Chairman S.W. Essex Div. B.M.A.

492 Lea Bridge Road, Leyton, N.E.

August 26, 1913.

